# Latexin deficiency attenuates adipocyte differentiation and protects mice against obesity and metabolic disorders induced by high-fat diet

**DOI:** 10.1038/s41419-022-04636-9

**Published:** 2022-02-24

**Authors:** Shuang Kan, Rong Li, Yanhui Tan, Fang Yang, Shaohua Xu, Lingzhu Wang, Lijun Zhang, Xuchen Sun, Xuanming Chen, Yuting Yang, Wei Shu, Huaibin Wan, Zheng-Feng Chen, Hong Liang, Ming Chen

**Affiliations:** 1 State Key Laboratory for Chemistry and Molecular Engineering of Medicinal Resources, Collaborative Innovation Center for Guangxi Ethnic Medicine, School of Chemistry and Pharmacy, Guangxi Normal University, Guilin, 541004 Guangxi P. R. China; 2 School of Environment and Resources, Guangxi Normal University, Guilin, 541004 Guangxi P. R. China; 3 The Guangxi Key Laboratory of Environmental Exposomics and Entire Lifecycle Heath, College of Biotechnology, Guilin Medical University, Guilin, 541004 Guangxi P.R. China; 4 Heyuan Research Center for Cardiovascular Diseases, Department of Cardiology, the Fifth Affiliated Hospital of Jinan University, Heyuan, 517475 Guangdong P.R. China

**Keywords:** Metabolic disorders, Mechanisms of disease

## Abstract

Obesity is a risk factor for many chronic diseases, and is associated with increased incidence rate of type 2 diabetes, hypertension, dyslipidemia and cardiovascular diseases. Adipocyte differentiation play critical role during development of obesity. Latexin (LXN), a mammalian carboxypeptidase inhibitor, plays important role in the proliferation and differentiation of stem cells, and highlights as a differentiation-associated gene that was significantly downregulated in prostate stem cells and whose expression increases through differentiation. However, it is unclear whether LXN is involved in adipocyte differentiation. The aim of this study was to evaluate the role of LXN on adipocyte differentiation, as well as its effects on high fat-induced obesity and metabolic disorders. In this study, we determine the expression of LXN in adipose tissue of lean and fat mice by Western blot, qPCR and immunohistochemistry. We found that LXN in fat tissues was continuous increased during the development of diet-induced obesity. We fed wild-type (WT) and LXN^−/−^mice with high-fat diet (HFD) to study the effects of LXN on obesity and related metabolic functions. We found that mice deficient in *LXN* showed resistance against high-fat diet (HFD)-induced obesity, glucose tolerance, insulin tolerance and hepatic steatosis. In vitro studies indicated that LXN was highly induced during adipocyte differentiation, and positively regulated adipocyte differentiation and adipogenesis in 3T3-L1 cells and primary preadipocytes. Functional analysis revealed that the expression of LXN was positively regulated by mTOR/RXR/PPARɤ signaling pathway during the differentiation of adipocytes, while *LXN* deletion decreased the protein level of PPARɤ in adipocyte through enhancing FABP4 mediated ubiquitination, which led to impaired adipocyte differentiation and lipogenesis. Collectively, our data provide evidence that *LXN* is a key positive regulator of adipocyte differentiation, and therapeutics targeting LXN could be effective in preventing obesity and its associated disorders in clinical settings.

## Introduction

Obesity is a risk factor for many chronic diseases. Obesity and associated comorbidities, such as type 2 diabetes, non-alcoholic fatty liver disease (NAFLD), hypertension, heart diseases and stroke, represent the most common health risks worldwide [1–3], and becoming one of the growing fundamental problem of human diseases in developing and underdeveloped countries. It is estimated that more than 4 million people die from overweight or obesity each year, and this figure is on the rising [4]. The prevalence of obesity has become a major burden on the global health system. Hence, the understanding of the molecular events in process of obesity is of growing importance.

Adipose tissue is a complex organ regulating energy balance, which is mainly composed of adipocytes surrounded by fibroblasts, fibroblastic preadipocytes, endothelial cells and immune cells [5]. Differentiation of preadipocytes into mature adipocytes is one of the important mechanisms of adipose tissue formation and obesity. During the development of obesity, adipose tissue expands through an increase in the size of preexisting adipocytes as well as through the formation of new adipocytes from preadipocytes resulting in an increased number of adipocytes [6]. In this regarding, mounting evidences suggest that differentiation of adipocytes from mesenchymal precursors is an important process for maintaining functional adipose tissues [7, 8]. Therefore, regulation of preadipocyte differentiation may be an effective way to treat obesity and related metabolic diseases.

Multiple signaling pathways and mediators have been described to regulate adipocyte differentiation such as bone morphogenetic protein (BMP) signaling, mTOC signaling and PPARγ/RXRα [9–15]. Among them, PPARγ as the dominant regulator of adipogenic differentiation was well studied. The expression of PPARγ is not only induced early during adipocyte differentiation but also continues at high level in mature adipocytes, suggesting that PPARγ may also play have important role in fully differentiated adipocytes [16]. Latexin (LXN), a mammalian carboxypeptidase inhibitor, was originally identified in the lateral neocortex of rats and widely expressed in tissues and cells including intestines, hematopoietic and lymphoid organs [17, 18]. Previous studies showed that LXN plays an important physiological role in stem cells proliferation and differentiation. For example, Liang et al. identified *LXN* as a stem cell regulatory gene with expression that negatively correlates with haematopoietic stem cell (HSC) number variation in different mouse strains [19], and they further reported that ablation of *LXN* enhanced long-term repopulating capacity and survival of HSCs through regulating the expression of *Thbs1* [20]. Kadouchi et al. reported that the expression of LXN is induced by BMP-2 in mesenchymal cells during chondrocyte and osteoblast differentiation, suggesting the important role of *LXN* in the cell differentiation [21, 22]. Oldridge et al. reported that *LXN* is a differentiation-associated gene that are highly significantly downregulated in prostate stem cells and whose expression increases through differentiation [23]. However, it is unclear whether *LXN* is involved in adipocyte differentiation.

In the present study, we examined the roles of *LXN* in adipocyte differentiation and obesity in mice. We show that *LXN*-deficient mice have reduced HFD-induced obesity and improved glucose tolerance and insulin sensitivity. We demonstrate that LXN is upregulated by mTOR/RXR/PPARɤ axis during adipocytes differentiation, and *LXN* deletion decreases the level of PPARɤ in adipocyte through enhancing FABP4 mediated ubiquitination.

## Results

### Expression of LXN is upregulated in adipose tissue of mice by body adiposity

**Fig. 1:**
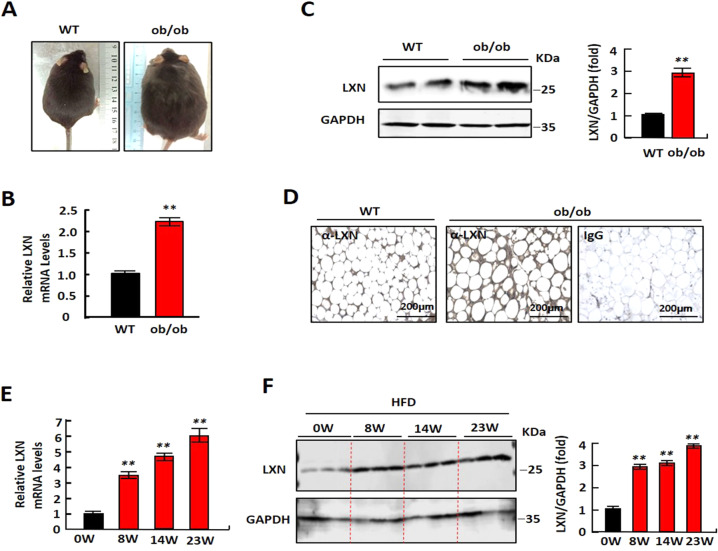
Enhanced expression of LXN in obese mice. **A** Representative pictures for WT and ob/ob mice after feeding with normal diet (ND) for 16 weeks. **B**, **C** QPCR (B) and Western blot (C) analysis for LXN in the subcutaneous adipose tissue of *WT* and *ob/ob* mice. The bar graphs showing the results of all animals examined. Data are mean ± SD. **P < 0.01 vs. WT. **D** Immunohistological analysis of LXN expression in adipose tissues of *WT* and *ob/ob* mice. Scale bars, 200 μm. **E**, **F** 8-week-old male C57/B6 mice (*n* = 6) were fed with high fat-diet (HFD) for additional 8, 14 or 23 weeks, then adipose tissue were harvested. LXN was determined by QPCR (**E**) and western blot (**F**). The bar graph shows quantification of LXN level in all animals examined. **P < 0.01 vs. 0 W.

We first analyzed the expression of *LXN* in obese rat or mice fat tissues based on GEO profile database (GEO profiles: https://www.ncbi.nlm.nih.gov/geoprofiles). We found that *LXN* was significantly upregulated in epididymal adipose tissue of diet-induced obese rat (GEO 44671117) (Fig. S1A). In mice, we found that mice with high weight gain had more *LXN* expression in adipose tissue compared with mice with low body weight gain (GEO 30162583) (Fig. S1B). We next examined the protein level of LXN in lean and obese mice. We constructed leptin receptor mutant mice (*ob/ob*) with C57/BL6 background. Compared with C57/BL6 mice (WT), *ob/ob* mice fed with normal diet (ND) gained more weight gain (Fig. 1A). The protein and mRNA level of LXN were much higher in adipose tissues in *ob/ob* mice than those in WT mice (Fig. 1B, C, D). When C57/BL6 mice were induced to develop obesity by feeding with high-fat diet (HFD), the expression of LXN in adipose tissues was significantly upregulated after 8-23 weeks of HFD feeding (Fig. 1E, F). These data suggest that the expression of LXN is positively correlated with obesity in mice.

### Mice deficient in *LXN* are protected from HFD-induced obesity

**Fig. 2:**
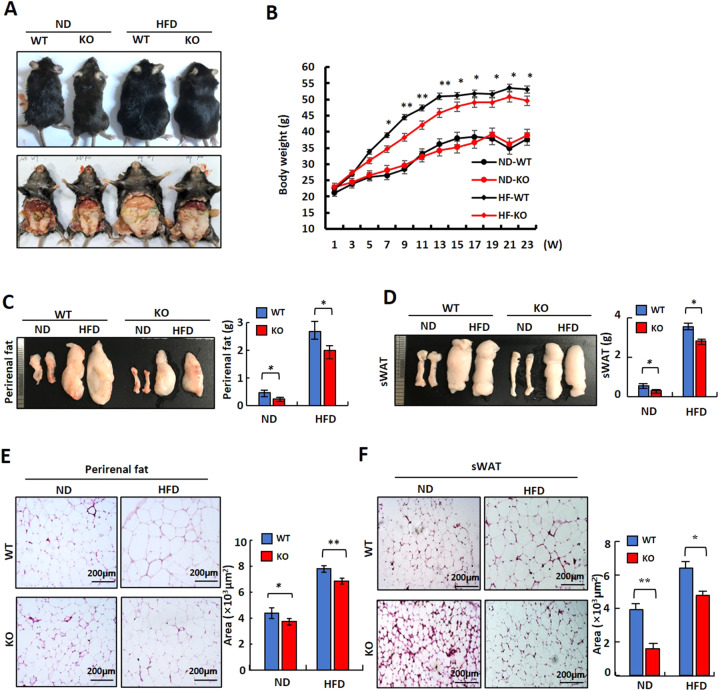
Reduced adiposity in LXN-deficient mice. **A** Representative pictures for *WT* and *LXN*^*−/−*^ mice after feeding with HFD or ND for 23 weeks. **B** Comparison of body weight changes between *WT* and *LXN*^*−/−*^ mice during the course of ND or HFD induction (*n* = 8). ND-WT, WT mice fed with ND; ND-KO, *LXN*^*−/−*^ mice fed with ND; HF-WT, WT mice fed with HFD; HF-KO, *LXN*^*-/-*^ mice fed with HFD. **C**, **D** Representative pictures for perirenal adipose tissue (**C**) and subcutaneous adipose tissue (**D**) collected from *WT* and *LXN*^*-/-*^ mice after 23 weeks of HFD or ND feeding, and analysis of the weight. **E**, **F** Representative pictures for H&E-stained perirenal adipose sections (**E**) and subcutaneous adipose sections (**F**) collected from *WT* and *LXN*^*-/-*^ mice after 23 weeks of HFD or ND feeding. Scale bar = 200 μm. Mean adipocyte size was calculated. All results were presented as mean ± SD, and *P* values were calculated with use of Student *t* test. *P < 0.05, and **P < 0.01.

We next sought to address the impact of *LXN* on the development of obesity. For this purpose, 8-week-old *LXN*^*−/−*^ and control littermates were fed with HFD for 23 weeks. We found that overall body weight gain was higher in HFD-induced WT mice than that of *LXN*^*-/-*^ mice (Fig. 2A, B). Importantly, the lower body weight in HFD-fed *LXN*^*-/-*^ mice was predominantly featured by the reduction of white adipose tissue (WAT) mass such as the perirenal adipose tissue (WT = 2.68 ± 0.44 g vs. KO = 1.99 ± 0.55 g; *P* < 0.05) and subcutaneous white adipose tissue (sWAT) (WT = 3.53 ± 0.36 g vs. KO = 2.91 ± 0.28 g; *P* < 0.05) (Fig. 2C, D). Consistent with these observations, *LXN*^*−/−*^ mice displayed significantly smaller size for adipocytes in perirenal adipose tissues and sWAT than those of wild-type mice fed with either ND or HFD (Fig. 2E, F). Collectively, these data provided evidence suggesting that *LXN*-deficient mice are resistant to HFD-induced obesity.

### *LXN* is induced during adipocyte differentiation, and *LXN* deficiency inhibits adipocyte differentiation and adipogenesis in vitro

**Fig. 3:**
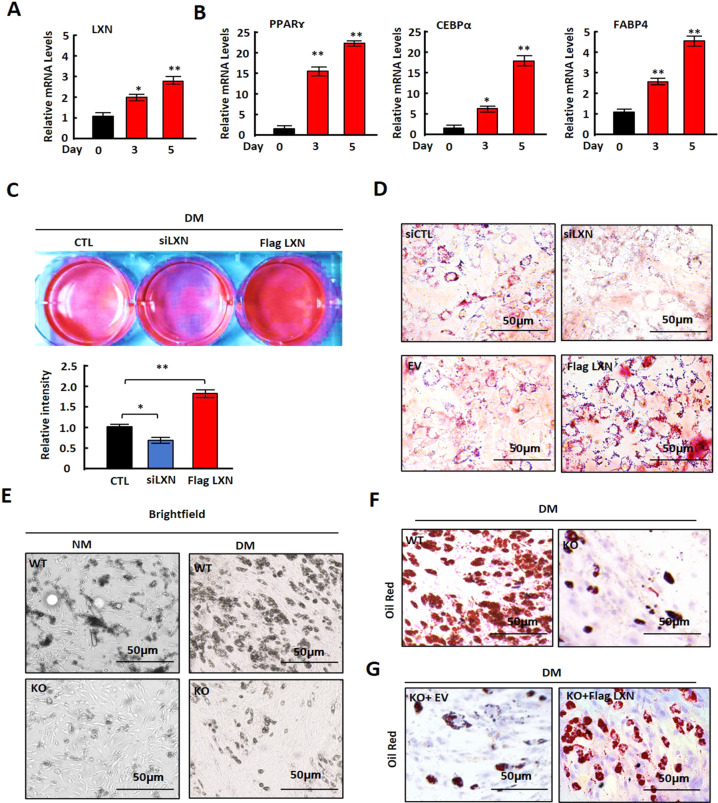
LXN deficiency inhibits adipocyte differentiation and adipogenesis in vitro. **A**, **B** 3T3-L1 cells were cultured with differentiation medium for 0,3 and 5 d, the mRNA level of LXN (**A**), PPARɤ, CEBPα and FABP4 (**B**) were determined by qPCR. **C**, **D** 3T3-L1 cells were transfected with LXN siRNA or flag-LXN plasmid. After 48 h, the cells were cultured with differentiation medium for 3 d. Cultured cells were subjected to Oil Red O staining (**C**) and imaged by EVOS microscope (**D**). EV empty vector, siCTL siRNA control, Scale bar = 50 μm. **E**, **F** WT and *LXN*^*−/*−^ preadipocytes (KO) were cultured with differentiation medium for 3 d (**E**). Cultured cells were subjected to Oil Red O staining (**F**). Scale bar = 50 μm. **G** *LXN*^*−/−*^ preadipocytes were transfected with flag-LXN plasmid or empty vector. The cells were cultured with differentiation medium for 3 d, and subjected to Oil Red O staining. Scale bar = 50 μm.

We confirmed the expression of *LXN* during the differentiation of 3T3-L1 cells. The qPCR analyses revealed that the mRNA level of *LXN* increased after induction during adipocyte differentiation (Fig. 3A). As expected, these genes associated with adipocyte differentiation, such as *PPARɤ*, *CEBPα* and *FABP4*, were also induced (Fig. 3B). We further evaluated the effect of LXN on adipogenesis of preadipocytes. We determined the effect of LXN on adipocyte differentiation by using 3T3-L1 cells and primary preadipocytes from mice sWAT. In 3T3-L1 cells, differentiation medium (DM)-induced adipogenesis was significantly inhibited when *LXN* knockdown, however, enhanced by ectopic expression of LXN, as evidenced by lipid droplets determined by Oil Red O staining (Fig. 3C, D). Primary preadipocytes were isolated from sWAT of *WT* and *LXN*^*−/−*^ mice. A significant reduction of adipocyte differentiation was observed in *LXN*^*−/−*^ preadipocytes as compared with *WT* control when induced by DM (Fig. 3E, F). In contrast, the adipogenic ability of *LXN*^*-/-*^ preadipocyte could be restored by ectopic expression of *LXN* (Fig. 3G). These data indicate that LXN positively regulates preadipocyte differentiation and adipogenesis, whereas *LXN* deficiency inhibits this process.

### RNA-seq analysis reveals that *LXN* depletion in preadipocytes down-regulates PPARn targeted genes

**Fig. 4:**
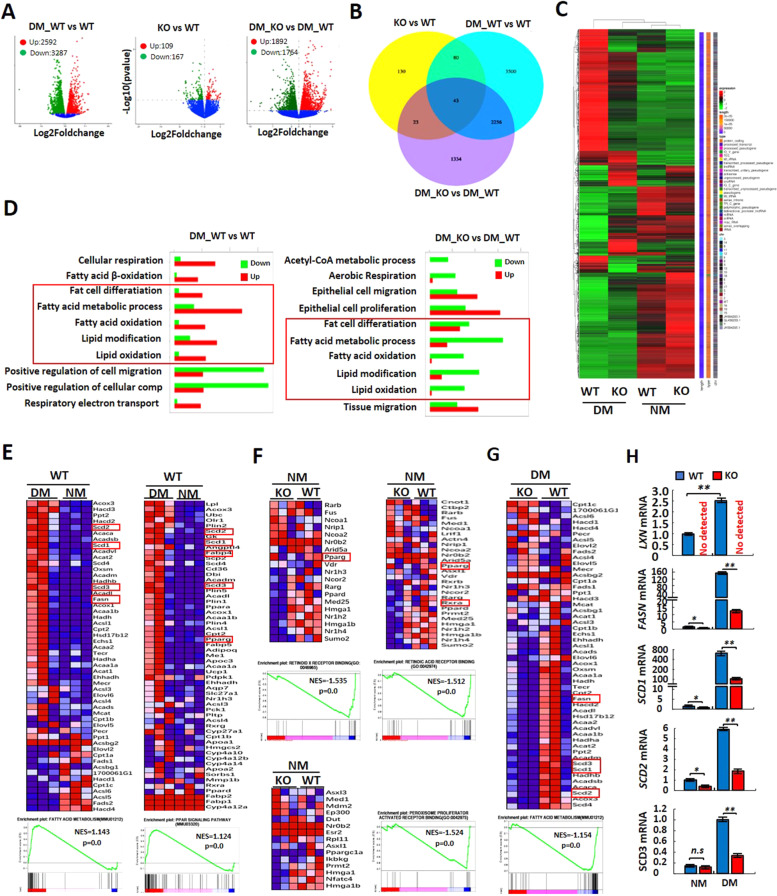
RNA sequencing reveals down-regulation of PPARɤ targeted genes in preadipocytes after deletion of LXN. **A** Volcano plot of differentially expressed genes (DGEs) in WT and KO preadipocytes cultured with normal medium (KO vs WT), WT preadipocytes cultured with normal medium and differentiation medium (DM_WT vs WT) and WT or KO preadipocytes cultured with differentiation medium (DM_KO vs DM_WT). **B** The overlapping genes identified in different experimental groups. **C** DEGs identified by RNA-seq were presented in Heatmap. **D** GO enrichment analysis of DEGs in DM_WT vs WT group (left) and DM_KO vs DM_WT group (right). **E**–**G** Heatmap shows the GSEA of representative KEGG pathway in WT preadipocytes cultured with differentiation medium or normal medium (**E**), WT and KO preadipocytes cultured with normal medium (**F**) and differentiation medium (**G**). **H** Real-time PCR results for analysis of LXN and PPARɤ targeted genes in WT or KO preadipocytes under normal medium or differentiation medium. All results were presented as mean ± SD. *P < 0.05, and **P < 0.01, ns: no significance.

To comprehensively understand the effect of *LXN* deficiency on adipocyte differentiation, we performed RNA-seq analysis with *WT* and *LXN*^*−/*−^ preadipocytes. Preadipocytes from *WT* and *LXN*^*−/−*^ mice were treated with NM or DM for three days. RNA-seq revealed that *LXN* ablation induced significant transcriptional changes of preadipocyte under normal medium or differentiation medium treatment (Fig. 4A, B), and suggested that *LXN* inhibition leads to the dysregulation of different subsets of transcriptional in the absence or presence of differentiation medium (Fig. 4C). To identified biological processes affected by *LXN* deficiency, we performed functional annotation by gene ontology (GO). We focused on the NM- and DM-induced *WT* preadipocytes (DM_WT vs. WT) and DM-induced *WT* and *LXN*^−*/−*^ preadipocytes (DM_WT vs DM_KO). Our data show that those upregulated genes were enriched for fat cell differentiation, fatty acid metabolic process, fatty acid oxidation, lipid modification and lipid oxidation when *WT* preadipocytes were treated with DM for three days; whereas these gene groups were mostly downregulated in DM-induced *LXN*^*-/-*^ preadipocytes as compared with DM-induced *WT* preadipocytes (Fig. 4D). Gene set enrichment analysis (GSEA) further confirmed the upregulation of fatty acid metabolism and PPARɤ signaling signatures, including pro-adipogenesis genes (such as *Fasn, Scd1, Scd2, Scd3* and *Pparɤ*) in DM-induced *WT* preadipocytes (Fig. 4E); whereas, under normal medium, *LXN* deficiency did not affect the mRNA level of PPARɤ, but significantly decreased retinoid x receptor (RXR) binding, retinoic acid receptor (RAR) binding and peroxisome proliferator activated receptor (PPAR) binding activities (Fig. 4F), indicating the important role of *LXN* in the RXR/PPAR mediated proliferation and differentiation of preadipocytes. Importantly, GSEA revealed that DM-induced fatty acid metabolism was dramatically inhibited in *LXN*^*−/−*^ preadipocytes (Fig. 4G), which was characterized by decreasing of PPARɤ target genes such as *Fasn* [24, 25] *Scd1* [25]*, Scd2* [26] and *Scd3*, compared with DM-induced *WT* preadipocytes (Fig. 4H). Overall, these data indicate that *LXN* deficiency inhibits adipocytes differentiation by reducing PPARɤ target genes.

### LXN is upregulated by activation of mTOR/RXR/PPAR/ signaling pathway during adipocytes differentiation and adipogenesis

**Fig. 5:**
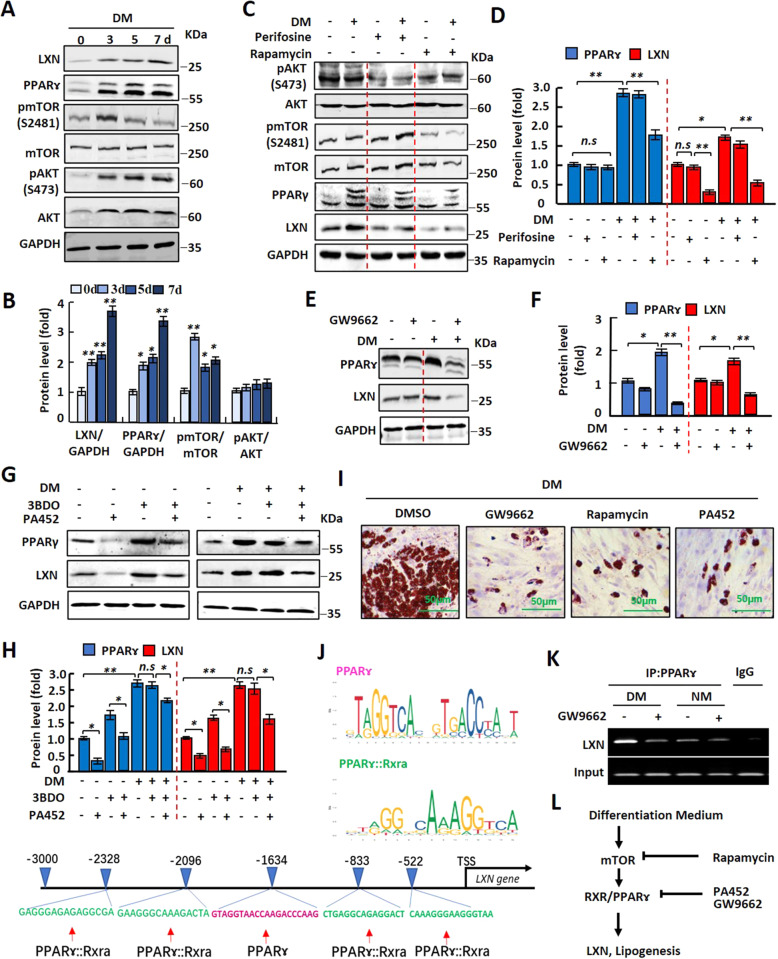
mTOR/RXR/PPARɤ signaling pathway is required for the upregulation of LXN during adipogenesis. **A**, **B** 3T3-L1 cells were cultured in differentiation medium for 0, 3, 5, 7 d. Cell extracts were subjected to Western blot analysis with the indicated antibodies (**A**) and then quantified and normalized (**B**). *P < 0.05, and **P < 0.01 vs. 0 d. **C**, **D** 3T3-L1 cells were pre-treated with 10µmol/L perifosine (AKT inhibitor) or 20nmol/L rapamycin (mTOR inhibitor) for 12 h. After that, the cells were cultured with normal medium or differentiation medium for 3 d. The cell extracts were then subjected to Western blot analysis with the indicated antibodies (**C**), and the relative protein levels of PPARɤ and LXN were assessed (**D**). **E**, **F** 3T3-L1 cells were pre-treated with 10 µmol/L GW9662 (PPARɤ antagonist) for 2 h, and then cells were cultured with normal medium or differentiation medium for 3 d. The cell extracts were then subjected to Western blot analysis with the indicated antibodies (**E**), and the relative protein levels of PPARɤ and LXN were assessed (**F**). **G**, **H** Normal medium or differentiation medium-cultured 3T3-L1 cells were treated with 20 µmol/L 3BDO (mTOR agonist) for 12 h followed by treatment with 20 µmol/L PA452 (RXR antagonist) for an additional 60 h. to assess PPARɤ and LXN protein levels. The cell extracts were then subjected to Western blot analysis with the indicated antibodies (**G**), and the relative protein levels of PPARɤ and LXN were assessed (**H**). **I** 3T3-L1 cells were cultured in differentiation medium with GW9662, rapamycin or PA452, respectively, for 3 d. Cultured cells were subjected to Oil Red O staining. Scale bar = 50 μm. **J** Prediction of PPARɤ and PPARɤ::Rxra binding sites in *LXN* promoter region (~3000 bp) by JASPAR CORE database (https://jaspar.genereg.net/). Pink, represents the predicted PPARɤ binding region; Green, represents the predicted PPARɤ::Rxra binding region; **K** 3T3-L1 cells cultured in normal medium (NM) and differentiation medium (DM) with or without GW9662 (PPARɤ antagonists) treatment for 3 d, and the binding activity of PPARɤ to *LXN* promoter in 3T3-L1 cells was determined by ChIP assay. **L** Schematic diagram to demonstrate the mechanism by which LXN was upregulated during preadipocyte differentiation. All results were presented as mean ± SD. *P < 0.05, and **P < 0.01, ns: no significance.

The expression of LXN was upregulated in obese mice and could be induced by high-fat diet (Fig. 1, Supplementary Fig. S1). We then want to ask what the mechanism underline high-fat diet induces the expression of LXN. To this end, we assessed the expression of LXN during differentiation of preadipocyte by cultured cells with differentiation medium (DM) for 0, 3, 5, 7 days. We found that the LXN protein level began to increase after 3 days of DM induction and reached the maximum after seven days. As expected, the PPARɤ protein level was continued to rise during DM induction. Treatment of preadipocytes with DM activates mTOR, however, has little effect on the activation of AKT, although it increases the protein level of AKT (Fig. 5A, B). To expound the pathways underline LXN is upregulated during adipogenesis, DM-induced preadipocytes were treated with perifosine (AKT inhibitor) and rapamycin (mTOR inhibitor), respectively. Notably, the upregulation of LXN and PPARɤ in DM-induced preadipocytes were markedly inhibited by treatment with mTOR inhibitor rapamycin, rather than AKT inhibitor (Fig. 5C, D), indicating the critical role of mTOR for upregulation of LXN and PPARɤ in DM-induced preadipocytes. Furthermore, treatment with PPARɤ inhibitor GW9662 significantly inhibited the expression of LXN in DM-induced preadipocytes (Fig. 5E, F), indicating PPARɤ is a positive regulator of LXN expression and is located downstream of mTOR signal. Interestingly, the increased LXN by mTOR agonist 3BDO was reversed when RXRα antagonists PA452 present (Fig. 5G, H), indicating that RXRα:PPARɤ, a common heterodimer for regulating the expression of adipocyte genes, contributed to LXN expression. Oil Red O-staining of DM-induced preadipocytes further proved that inhibitors like GW9662, rapamycin and PA452 significantly suppress adipogenesis (Fig. 5I). We further analyzed the transcription factor binding site of ~3000 bp (-2973-1) upstream of the TSS site of mouse *LXN* gene (gene ID 17035) by using Jaspar online tool [27]. Total five putative sites were predicted with relative profile score threshold 80%, among them, 1 PPARɤ binding site and 4 PPARɤ::RxRa binding sites were predicted (Fig. 5J, and Supplementary Table S1). The binding activity of PPARɤ to the promoter region of *LXN* gene was confirmed in 3T3-L1 cells by ChIP experiment (Fig. 5K). So far, we identified the molecular signaling pathway involved in regulating the expression of LXN in preadipocytes (Fig. 5L), and strongly suggest that the expression of LXN is positively regulated by mTOR/RXR/PPARɤ signaling pathway during the differentiation of preadipocytes into adipocytes.

### *LXN* deficiency in preadipocyte decreases the stability of PPARɤ by enhancing FABP4 mediated ubiquitination

**Fig. 6:**
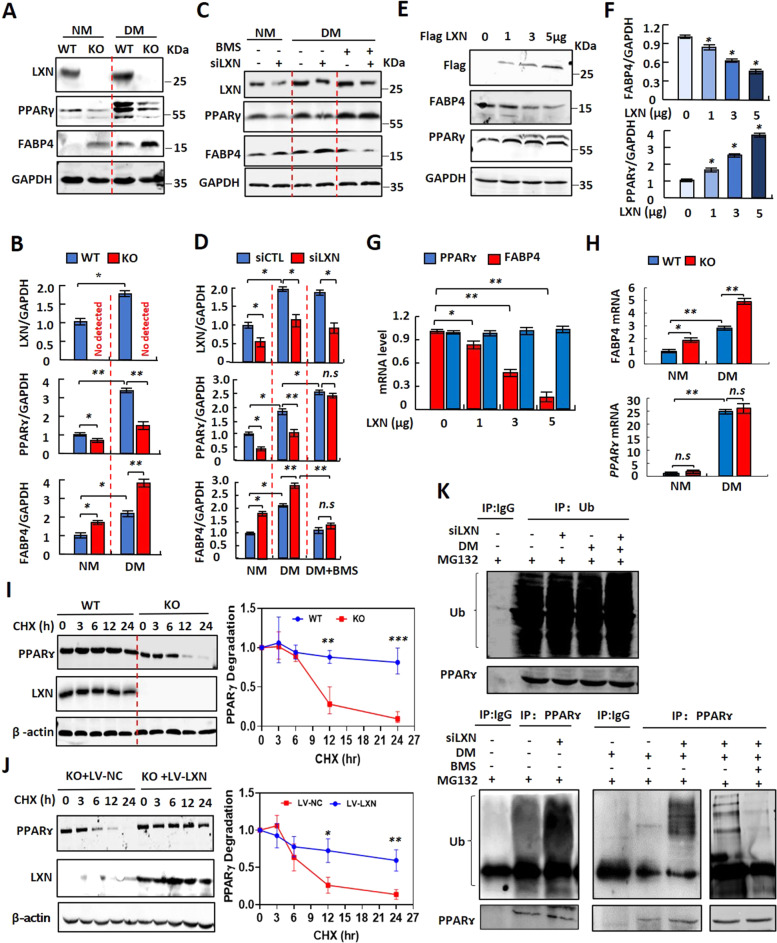
LXN negatively regulates PPARɤ ubiquitination by inhibiting FABP4 expression. **A**, **B** Primary preadipocytes isolated from *WT* and *LXN*^*−/−*^ mice were cultured with normal medium (NM) or differentiation medium (DM) for three days. Cell extracts were subjected to Western blot analysis with the indicated antibodies (**A**), and the relative protein levels of LXN, PPARɤ and FABP4 were assessed (**B**). **C, D** 3T3-L1 cells were transfected with scramble siRNA or LXN siRNA. Twelve hours later, the medium was changed, and the cells were cultured in the differentiation medium containing 20 µmol/L BMS (FABP4 inhibitor) for 3 days. The cell extracts were then subjected to Western blot analysis with the indicated antibodies (C), and the relative protein levels of LXN, PPARɤ and FABP4 were assessed (D). **E-G** Normal medium-cultured 3T3-L1 cells were transfected with 0, 1, 3, 5 µg Flag-LXN plasmid, respectively, for 48 h. The cell extracts were then subjected to Western blot analysis with the indicated antibodies (E), and relative quantification of FABP4 and PPARɤ were presented (F). The relative mRNA levels of FABP4 and PPARɤ were determined by qPCR (G). **H** qPCR determine the relative mRNA level of FABP4 (left) and PPAR ɤ (right) in *WT* and *LXN*^*-/-*^ primary preadipocytes under normal medium or differentiation medium condition. **I** *WT* and *LXN*^*-/-*^ primary preadipocytes were treated with CHX (350 mmol/L) and harvested at the indicated times after CHX addition. Immunoblots of cell extracts with antibodies directed against PPARɤ, LXN, and β-actin are shown (left). Half-life analysis of PPARɤ protein, relative to time 0 (right). **J** Overexpression of LXN by lentivirus in *LXN*^*-/-*^ primary preadipocytes followed by CHX treatment for the indicated times. Immunoblots of cell extracts with antibodies directed against PPARɤ, LXN, and β-actin are shown (left). Half-life analysis of PPARɤ protein, relative to time 0 (right). **K** 3T3-L1 cells were transfected with scramble siRNA or LXN siRNA for 48 h. After that the cells were treated with 20 µmol/L BMS for 12 h, and then the cells were cultured with differentiation medium. After 48 h, the cells were treated with MG132 (0.1 µmol/L) for another 12 h. Cell lysates were immunoprecipitated with an anti-ubiquitin or anti-PPARɤ antibody, and the immunoprecipitates were immunoblotted with antibodies directed against ubiquitin or PPARɤ. All results were presented as mean ± SD. *P < 0.05, and **P < 0.01, *** *P* < 0.001, ns: no significance.

PPARɤ is recognized as a central coordinator of the adipogenic program, and acts as a direct activator of most adipocyte specific genes [15, 28, 29]. We observed that loss of LXN ameliorated HFD-induced obesity in mice and inhibited adipogenesis of adipocytes in vitro (Fig. 2 and Fig. 3), which prompted us to ask whether LXN deletion affects the expression of PPARɤ. To this end, WT and LXN^-/-^ preadipocytes were treated with normal medium (NM) or differentiation medium (DM) for 3 days. As expected, preadipocytes cultured with DM significantly increase the protein level of PPARɤ and fatty acid binding protein (FABP4). However, LXN deletion dramatically attenuates the protein level of PPARɤ, but enhances that of FABP4, compared with WT preadipocytes under both NM and DM-treated conditions (Fig. 6A, B). Similar story occurred in 3T3-L1 cells treated with siLXN. Interestingly, the decrease in PPARɤ caused by LXN knockdown was restored when cells were treated with FABP4 inhibitor BMS (Fig. 6C, D). FABP4 is one of major target gene of PPARɤ, and FABP4 was induced by PPARɤ almost exclusively in adipocytes [30, 31]. Garin–Shkolnik et al. reported that FABP4 decreases PPARɤ protein level by accelerating its ubiquitination, suggesting a negative feedback loop between PPARɤ and FABP4 in adipocytes [32]. Therefore, our results demonstrate that inhibition of LXN in preadipocytes elevates FABP4 level, which acts as a negative regulator of PPARɤ, thus suggesting a positive feedback loop exists between LXN and PPARɤ in adipocytes.

To strengthen the observations, we further determined the effect of LXN on the expression of PPARɤ and FABP4. To this end, normal medium-cultured 3T3-L1 cells were transfected with Flag-LXN plasmid. We found that the protein level of PPARɤ was increased in a dose-dependent manner with LXN, while the protein level of FABP4 was decreased with the enhanced expression of LXN (Fig. 6E, F). QPCR showed that ectopic expression of LXN significantly decreased *FABP4* mRNA level in 3T3-L1 cells; however, had no effect on the level of *PPARɤ* mRNA (Fig. 6G). In contrast, we observed an increase of *FABP4* mRNA in *LXN* deficient primary preadipocytes under both NM and DM-treated conditions (Fig. 6H, up). Although DM induced an increase in *PPARɤ* mRNA, no difference was observed between *WT* and *LXN*^*-/-*^ preadipocytes absence or presence of differentiation medium (Fig. 6H, bottom). These results clarify that LXN regulates PPARɤ expression at the protein level rather than at the mRNA level.

We therefore examine the effect of *LXN* knockout on the stability of PPARɤ protein. Protein half-life analysis showed that PPARɤ protein became more unstable in *LXN-*deficient preadipocytes as compared with *WT* preadipocytes (Fig. 6I). However, *LXN*^*-/-*^ preadipocytes ectopic expression of LXN by infected with lentivirus dramatically enhances PPARɤ stability (Fig. 6J), further suggesting that LXN positively regulates the stability of PPARɤ protein. We then sought to confirm the ubiquitination state of PPARɤ. As shown in Fig. 6K, although *LXN* knockdown had little effect on the overall ubiquitination level of 3T3-L1 cells, *LXN* knockdown significantly increased PPARɤ ubiquitination under normal medium condition. Differentiation medium treatment almost inhibited PPARɤ ubiquitination; however, this effect was reversed when *LXN* was knockdown. It was interestingly noted that the ubiquitination of PPARɤ caused by *LXN* knockdown is attenuated when cells were pre-treated with FABP4 inhibitor BMS, indicating that FABP4 indeed mediated the decrease of PPARɤ in *LXN*^*-/-*^ adipocytes.

### *LXN* deficiency improves HFD-induced metabolic disorders in mice

**Fig. 7:**
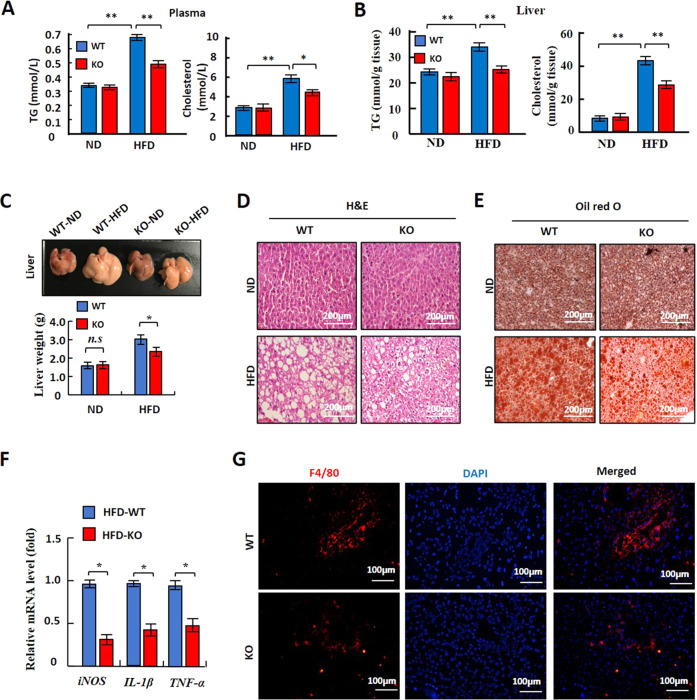
LXN-deficient mice are resistant to HFD-induced hepatic steatosis. **A**, **B** Results for TG and cholesterol levels in plasma (**A**) and liver (**B**) of *WT* and *LXN*^*-/-*^ mice fed a ND or HFD for 23 weeks (*n* = 8). **C** Comparison of liver weight. Top: Representative pictures for livers collected from *WT* and *LXN*^*−/−*^ mice. Bottom: Results for liver weight (*n* = 8). **D**, **E** Representative results for H&E staining (**D**) and Oil Red O staining (**E**) of liver sections. Scale bar = 200 μm. **F** Real-time PCR results for analysis of inflammation marker genes in liver (*n* = 8). **G** Representative pictures for immunofluorescence staining of F4/80 of liver sections. Scale bar = 100 μm. All results were presented as mean ± SD, and *P* values were calculated with use of Student *t* test. *P < 0.05, and **P < 0.01, ns: no significance.

Metabolic disorder is a common complication of obesity. Since *LXN* deficiency can alleviate obesity induced by high-fat diet, we want to know whether *LXN* deficiency can also improve obesity complications, such as hepatosteatosis and insulin resistance. We challenged WT and *LXN*^*-/-*^ mice with HFD feeding for a 23-week period. The concentration of triglycerides (TG) and cholesterol in plasma and liver tissue were determined. No significant difference in plasma triglyceride and cholesterol was observed between *WT* and *LXN*^*−/−*^ mice on normal diet. However, mice deficient in *LXN* were manifested by the significantly lower levels of plasma TG and cholesterol than their littermate controls after 23 weeks of HFD induction (Fig. 7A), and the significantly lower levels of TG and cholesterol were noted in liver in *LXN*^*-/-*^ mice under HFD, as well (Fig. 7B). In line with these observations, hepatic steatosis was detected in HFD-fed *WT* mice as featured by the significant change of liver color and weight as compared with that of *LXN*^*-/-*^ mice (Fig. 7C). Histologic analysis of the liver from *WT* mice on HFD showed macrovesicular hepatic steatosis as evidenced by a fatty liver populated with abundant large vacuolar lipid droplets, whereas the liver of *LXN*^*−/−*^ mice had few lipid droplets, as determined by H&E and Oil Red O staining (Fig. 7D, E). Moreover, mRNA expression levels of inflammatory markers (such as iNOS, IL-6 and TNF-α) (Fig. 7F) and the immunofluorescence staining (F4/80) supported the histological evidence for reduced inflammation in the livers of *LXN*^*-/-*^ mice (Fig. 7G).

**Fig. 8:**
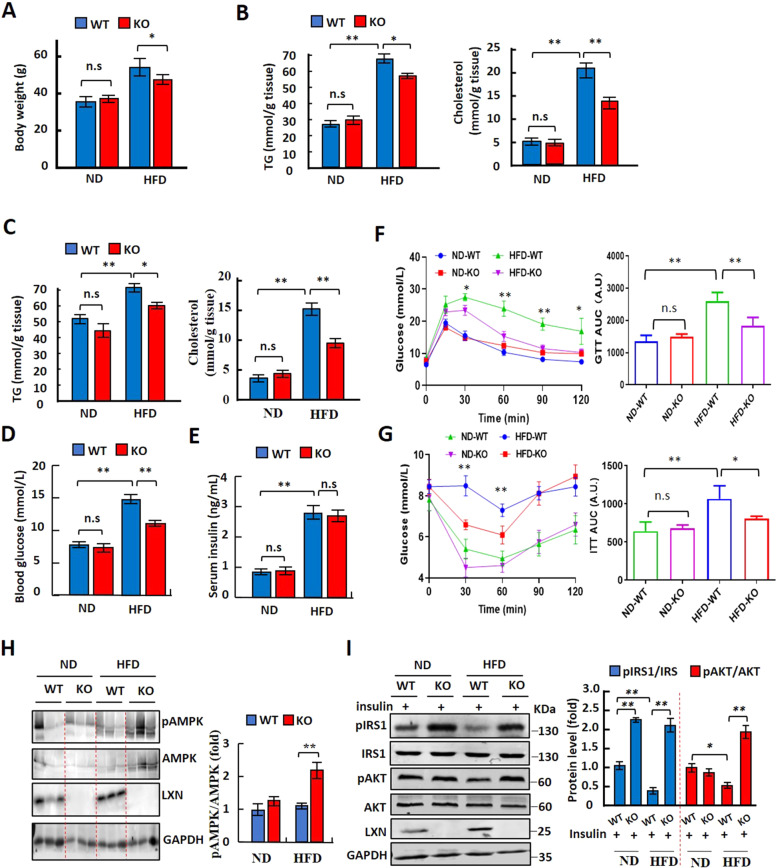
LXN deficiency ameliorates HFD-induced insulin resistance and glucose intolerance. **A** Body weight in *WT* and *LXN*^*−/−*^ mice fed a ND or HFD for 16 weeks (*n* = 8). **B**, **C** Results for TG and cholesterol levels in perirenal adipose tissue (**B**) and sWAT (**C**) of *WT* and *LXN*^*-/-*^ mice fed a ND or HFD for 16 weeks (*n* = 8). **D**, **E** Analysis of fasting plasma glucose (**D**) and insulin (**E**) levels. All mice were fasted for 12 h before the analysis. **F** Results for intraperitoneal glucose tolerance tests (GTT) (left) and area under the curve (AUC) for the blood glucose levels (right). **G** Results for intraperitoneal insulin tolerance tests (ITT) (left) and AUC for the blood glucose levels (right). For the GTT, fasting overnight mice were gavage fed with a 2 mg glucose/g body wt glucose load. For the ITT, mice fasted for 6 h were intraperitoneally injected with 0.5 U insulin/kg body wt using an insulin syringe. ND-WT, WT mice fed with ND; ND-KO, *LXN*^*−/*−^ mice fed with ND; HF-WT, WT mice fed with HFD; HF-KO, *LXN*^*-/-*^ mice fed with HFD. **H** Western blot analysis for p-AMPK, total AMPK, and LXN in the sWAT. **I** Western blot analysis for p-IRS1 (Ser307), total IRS1, p-AKT (Ser473), and total AKT in the sWAT. Mice were fasted overnight and injected intraperitoneally with insulin (1U/kg body wt). Tissues were excised 15 min after injection for immunoblotting analyses. All results were presented as mean ± SD, and *P* values were calculated with use of Student *t* test. *P < 0.05, and **P < 0.01, ns: no significance.

A chronic HFD treatment would result in obesity-associated insulin resistance in mice [2, 6, 15]. We also performed glucose tolerance and insulin sensitivity tests to examine the effect of *LXN* deficiency on HFD-induced insulin resistance. We placed *LXN*^*−/−*^ and *WT* mice on a normal diet or HFD for 16 weeks. *WT* and *LXN*^*−/−*^ mice were fed with HFD, which resulted in different body weight gain of mice, and *LXN*^*−/−*^ mice gained lighter body weight (Fig. 8A) accompanied with the markedly lower triglyceride and cholesterol level in both perirenal adipose tissue and sWAT compared with *WT* mice on HFD condition (Fig. 8B, C). Interestingly, HFD-induced *LXN*^*-/-*^ mice exhibited a lower blood glucose compared with *WT* mice (Fig. 8D), but there was no significant difference in plasma insulin level (Fig. 8E), suggesting the lower plasma glucose levels are caused by increased insulin sensitivity.

We therefore carried out glucose tolerance and insulin sensitivity tests to determine the effects of *LXN* deficiency on glucose metabolism. Unlike their *WT* littermates, *LXN*^*-/-*^ mice were characterized by the significantly improved glucose tolerance (Fig. 8F) and insulin sensitivity (Fig. 8G). Importantly, Western blot analysis showed that *LXN* loss obviously promote the phosphorylation of AMPK in sWAT of HFD-induced mice (Fig. 8H). Furthermore, Western blot analysis of IRS1 and AKT, the two critical insulin-signaling molecules, revealed that sWAT from *LXN*^*-/-*^ mice injected with insulin was featured by the significantly increased phosphorylated AKT (p-AKT, Ser473) and IRS1 (p-IRS1, Ser307) as compared with their control counterparts following 16 weeks of HFD induction (Fig. 8I). Taken together, these results provided evidences suggesting that loss of *LXN* in mice improves HFD-induced metabolic dysfunction.

## Discussion

Here, we investigated the role of LXN in adipocytes differentiation and metabolic disorders in mice with diet-induced obesity. We found that LXN is upregulated in adipose tissue of mice by body adiposity, and mice deficient in *LXN* were protected from HFD-induced obesity. We showed that LXN was upregulated during adipocytes differentiation, and *LXN* deficiency resulted in the reduction of adipogenesis of 3T3-L1 cells and primary preadipocytes induced by differentiation medium. Mechanistically, we show that mTOR/RXR/PPARɤ signaling pathway mediated the increase of LXN during adipocytes differentiation. *LXN* deficiency in preadipocyte decreases PPARɤ protein level by enhancing FABP4 mediated ubiquitination and ultimately attenuates adipocyte differentiation and lipogenesis. Importantly, we reveal that *LXN* deficiency in mice improves obesity-associated liver steatosis, glucose tolerance and insulin resistance, indicating the potential benefits of *LXN* deficiency in the diet-induced pathogenesis of obesity and obesity-associated metabolic syndrome (Fig S2).

Adipocyte differentiation from adipose precursor cells has been shown to be regulated by mTOR and PPARɤ signaling pathway [12, 28, 29]. The involvement of mTOR signaling in adipogenesis and lipid metabolism has been well recognized in recent years [33]. In particular, a positive role of mTOR in adipogenesis has long been implicated by rapamycin inhibition of preadipocyte differentiation [34–36], and activation of PPARγ ameliorated the differentiation deficiency of the mTOR-null adipocytes [37]. We found that the upregulation of LXN in DM-induced preadipocytes were markedly inhibited by mTOR inhibitor rapamycin or PPARɤ inhibitor GW9662, suggesting mTOR and PPARɤ are responsible for regulating LXN expression during preadipocyte differentiation. Retinoic acid has been reported to induce LXN expression through RXR [23, 38]. In adipocytes, PPARγ functions as an obligate heterodimer with RXRs, which participates in the expression of genes related to adipocyte differentiation and lipogenesis [30, 39, 40]. RXR agonists has been reported to enhance the activity of PPARγ-RXR signaling pathway in vivo [41, 42]. We also found that the increased LXN by mTOR agonist 3BDO was reversed when RXRα antagonists PA452 present. Moreover, transcription factor binding sites analysis and ChIP experiments showed that there were PPARɤ or PPARɤ:RXRa binding sites in *LXN* promoter region. It can thus be convinced that activation of mTOR/RXR/PPARɤ signaling pathway might contribute to elevated LXN expression during adipocyte differentiation.

The expression of PPARγ not only is induced early during adipocyte differentiation but also continues at a high level in mature adipocytes suggests that PPARγ also have important functions in fully differentiated cells [29, 39]. Actually, the level of PPARɤ is tightly regulated during adipocyte differentiation. For example, Garin–Shkolnik et al. reported that PPARɤ induces FABP4 expression in adipocytes, while excessive FABP4 triggers proteasomal degradation of PPARɤ creating a negative feedback loop, which in turn inhibits PPARɤ functions and consequently reduces adipocyte differentiation [32]. Therefore, the net effect of FABP4 seems to be to inhibit PPARɤ [43, 44]. Our data show that *LXN* deletion in preadipocytes decreased PPARɤ protein level at basal and DM condition, but significantly increased FABP4 protein level accordingly. It is worthy of note that the decrease in PPARɤ protein caused by *LXN* deficiency was attenuated by FABP4 inhibitors BMS. Importantly, it seems that LXN regulates PPARɤ expression at the protein level rather than at the mRNA level, because *LXN* deficiency in preadipocyte increases the mRNA level of FABP4, however, has no effect on that of PPARɤ. Therefore, we speculate that LXN regulates the expression of FABP4 through an unknown way, thereby regulating the stability of PPARɤ.

The stability of PPARɤ in *WT* and *LXN*^*-/-*^ preadipocytes was assessed. We confirmed that *LXN*^*-/-*^ preadipocytes displayed decreased half-life of PPARɤ, while overexpression of LXN increased its stability. In line with this observation, the PPARɤ ubiquitination has been noted to be increased in *LXN* knockout 3T3-L1 cells, while FABP4 inhibitor BMS attenuates this effect. Altogether, these findings demonstrate a critical role of PPARɤ/RXR/LXN axis in adipocytes differentiation, and reveal that *LXN* deletion decreases the protein level of PPARɤ in adipocyte through enhancing FABP4 mediated ubiquitination. FABP4 was identified as a cytosolic protein strongly upregulated during differentiation of preadipocytes into adipocytes [45, 46]. As a marker for adipocyte differentiation, FABP4 expression is specifically induced by insulin and/or IGF-1 [47], dexamethasone and fatty acids [48], and occurs as a downstream target of PPARγ activation [49]. We found that *LXN* deletion decreased the level of PPARɤ protein, but significantly increased the expression of FABP4 both at mRNA and protein levels. These findings indicate that LXN regulates FABP4 expression at least partially is PPARɤ independent, however, the cause by which LXN attenuates FABP4 expression is currently unknown, and this work is being carried out in our laboratory.

In summary, we demonstrated evidence, for the first time, that LXN functions as a regulator of adipocytes differentiation and adipogenesis. LXN positively regulates PPARɤ stability by inhibiting FABP4 mediated ubiquitination. Therefore, loss of *LXN* attenuates adipocyte differentiation, lipogenesis, and thus provides protection for mice against HFD-induced obesity and metabolic dysfunction, which may lead to new insights on diet-induced obesity and identify LXN as a potential target for the treatment of obesity and its associated disorders.

## Materials and methods

Materials and Methods are available in the online-only Data Supplement.

## Supplementary information

Author list confirmation

Materials and Methods

Supplementary Figure and Figure Legends

Supplementary Tables

Uncropped western blot

Reproducibility checklist

## Data Availability

The data sets generated during the current study are available from the corresponding author on reasonable request.
