# Supplementary material for: Latexin deficiency attenuates adipocyte differentiation and protects mice against obesity and metabolic disorders induced by high-fat diet
Source: Cell Death Dis. 2022 Feb 24;13(2):175. doi: 10.1038/s41419-022-04636-9 (PMC8873487; doi:10.1038/s41419-022-04636-9)
Supplement: Supplementary file 1 — Author list confirmation [file 41419_2022_4636_MOESM1_ESM.docx]

**1 Confirmation letter from Shuang Kan**

**2 Confirmation letter from Rong Li**

**3 Confirmation letter from Yanhui Tan**

**4** **Confirmation letter from Fang Yang**

**5 Confirmation letter from Shaohua Xu**

**6 Confirmation letter from Lingzhu Wang**

**7 Confirmation letter from Lijun Zhang**

**
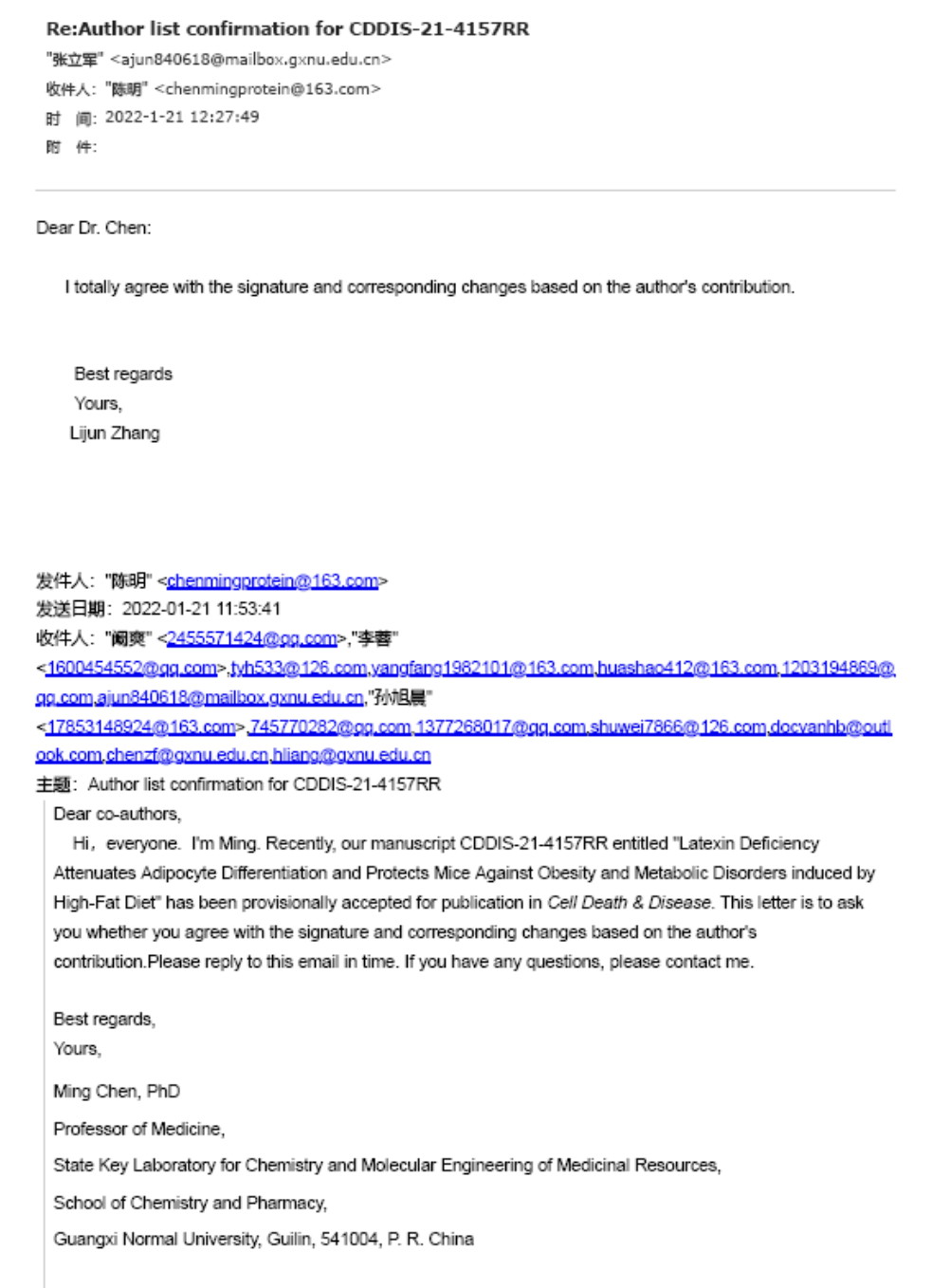
**

**8 Confirmation letter from Xuchen Sun**

**9 Confirmation letter from Xuanming Chen**

**10 Confirmation letter from Yuting Yang**

**11 Confirmation letter from Wei Shu**

**12 Confirmation letter from Huaibin Wan**

**13 Confirmation letter from Zhen-Feng Chen**

**14 Confirmation letter** **from Hong Liang**
