# Supplementary material for: Latexin deficiency attenuates adipocyte differentiation and protects mice against obesity and metabolic disorders induced by high-fat diet
Source: Cell Death Dis. 2022 Feb 24;13(2):175. doi: 10.1038/s41419-022-04636-9 (PMC8873487; doi:10.1038/s41419-022-04636-9)
Supplement: Supplementary file 2 — Materials and Methods [file 41419_2022_4636_MOESM2_ESM.docx]

**Supplementary material online**

Latexin Deficiency Attenuates Adipocyte Differentiation and Protects Mice Against Obesity and Metabolic Dysfunction induced by High-Fat Diet

Kan S., et al

**Materials and Methods**

**Plasmid, Small interfering RNA, Lentivirus and Reagents**

*LXN* was amplified by PCR using the primers containing BamHⅠ and EcoRⅠ sites (see Supplementary Table 1). *LXN* was sub-cloned into pFlag-CMV vector. Mouse LXN siRNA were purchased from Sigma (SASI_Mm01_00179145, ASSI_ Mm01_00179145_AS). siRNA was transfected into cells with Lipofectamine® RNAiMAX reagent (Invitrogen) according to the manufacturer’s recommendation. LXN expression lentivirus were packaged by Sangon Biotech Co., Ltd (Shanghai, China). Rapamaycin, GW9662, PA452, 3BDO, BMS-309403 and MG132 were purchased from MCE^®^ MedChemExpress (Shanghai, China).

**Mice**

*LXN* knockout *(*KO*; LXN^-/-^*) mice were maintained as F10 generations from *LXN*^+/-^ mice, which were generated and purchased from RIKEN BioResource Research Center (Japan). *ob/ob* mice with C57BL/6 background were purchased from Cavens laboratory animal Co., Ltd (Changzhou, China). Mice were randomly divided into two groups, and were fed a high fat-diet (HFD) (D12492, Research Diets, Inc. NJ, USA) or a normal chow diet (ND) as control to generate obesity model. Male mice of the same age were used in all experiments. All animal studies were conducted in accordance with the National Institutes of Health guidelines for the Care and Use of Laboratory Animals and approved by Medical and Animal Ethics Committee at Guangxi Normal University (Guilin, China).

**Cells**

3T3-L1 cells were purchased from ATCC. Primary preadipocytes were isolated from subcutaneous white adipose tissue (sWAT) of *WT* and *LXN^-/-^* mice accordingly. Cells were cultured in DMEM/F12 (Gibco) or differentiation medium (DMEM/F12 contains 10% FBS, 0.5 mM IBMX, 0.25 µM dexamethasone, 1 µg/mL insulin) in a humidified atmosphere of 5% CO_2_ at 37°C.

**Metabolic parameters**

For glucose tolerance test and insulin tolerance test, overnight fasted mice were gavage fed with a 2 mg glucose/g body weight glucose load. Blood samples were taken from the tail vein before and after glucose application at indicated time points, and glucose levels were measured using a glucometer (Accu-Chek Aviva). For insulin tolerance test, mice were fasted for 6 h, and then injected intraperitoneally with insulin (0.5U/kg body weight), and glucose levels were determined as described above. For examination of insulin signaling *in vivo*, the *WT* and *LXN^-/-^* mice fed HFD for 16 weeks were fasted overnight and then injected with 1 U/kg insulin or an equal volume of saline. Triglyceride assay kit (A110-1-1) and T-CHO assay kit (A111-1-1) were purchased from Jiancheng Bioengineering Institute (Nanjing, China). Mouse Insulin ELISA Kit (KA3812) was purchased from Amyjet Scientific (Wuhan, China). Triglyceride, cholesterol and Plasma insulin were determined according to the recommendations of these Kits.

**HE staining and immunohistochemistry**

Adipose tissues were fixed in 4% formaldehyde/PBS and maintained at 4°C until use. The fixed tissues were dehydrated and processed for paraffin embedding, and 4 µm sections were cut followed by staining with hematoxylin and eosin or indicated antibodies.

**Oil-Red-O staining**

Differentiated preadipocyte and 3T3-L1 cells were fixed with 4% Paraformaldehyde in PBS for 15 min and then washed 3 times in PBS for 10 min. Cells were washed for 30 s in 60% isopropanol and stained with Oil-Red-O (Solarbio) for 30 min and rinsed with 60% isopropanol for 1 min followed by water. Oil-Red-O-stained cells were directly imaged using an Invitrogen™ EVOS™ FL Auto2 microscopy (Thermo Fisher Scientific)

**Transcription factor (TF) binding site prediction**

Mouse *LXN* (gene ID 17035, span on NC_000069.7) was download from NCBI database (<https://www.ncbi.nlm.nih.gov/gene/?term=LXN>). Mouse *LXN* promoter about 3000 bp (-2973-1) upstream of TSS was extracted by NCBI Graphical Sequence Viewer (Version 3.44.1). TF sites were predicted by online tool Jaspar (<https://jaspar.genereg.net/>). The relative profile score threshold is 80%, and the results are listed in Supplementary Table S1.

**ChIP.** Formaldehyde-cross-linked chromatin was prepared from 3T3-L1 cells, and ChIP was performed using the SimpleChIP® Plus Enzymatic Chromatin IP Kit (Magnetic Beads #9005) from CST according to the manufacturer’s instructions. To calculate DNA enrichment in the ChIP assays, PCR was performed in a CFX96^TM^ Real-Time system (Bio-Rad) using the iQ SYBR Green supermix (Bio-Rad) and the primers for the *LXN* promoters (F:5’-ACTCAAGTCTGAAGATAATGGACTG-3; R:5’- GGAGTTAGAGATGACTGTGAGC-3’) were used.

**RNA extraction and qPCR**

Total RNAs were extracted from cells or tissues by using TRIzol Reagent (Life Technologies, Rockville, MD) according to the manufacturer’s instructions. qRT-PCR was performed on cDNA from 200 ng of total RNA by using cDNA Synthesis kit and SYBR® Green Master Mix Kit (Exqion). Samples were run on CFX96 Real-Time system (Bio-Rad). The sequences of the primer pairs are summarized in Supplementary Table S2.

**Western blotting**

Cells were lysed using RIPA buffer (25 mM Tris-HCl, 150 mM NaCl, 1% Nonidet P-40, 1% sodium deoxycholate, 0.1% SDS, pH 7.6). Protein samples were resolved by SDS-PAGE and transferred to nitrocellulose (BioRad). Blots were visualized on an Odyssey Imaging System (Licor). The antibodies for WB are listed in Supplementary Table S3.

**RNA-seq**

Total RNAs isolated from WT or *LXN^-/-^* primary preadipocytes using TRIzol reagent (Life Technologies, Rockville, MD). RNA integrity was assessed using the RNA Nano 6000 Assay Kit of the Bioanalyzer 2100 system (Agilent Technologies, CA, USA). The purified RNA samples, with RIN (RNA Integrity Number) over 8.0, determined by Agilent 2100 Bioanalyzer (Agilent, Waldbroon, Germany) were sequenced at Novogene corporation (Beijing, China). The clustering of the index-coded samples was performed on a cBot Cluster Generation System using TruSeq PE Cluster Kit v3-cBot-HS (Illumia) according to the manufacturer’s instructions. After cluster generation, the library preparations were sequenced on an Illumina Novaseq platform and 150 bp paired-end reads were generated. Differential expression analysis of two conditions/groups (two biological replicates per condition) was performed using the DESeq2 R package (1.16.1). Genes with an adjusted P-value <0.05 found by DESeq2 were assigned as differentially expressed. Gene Ontology (GO) enrichment analysis of differentially expressed genes was implemented by the cluster Profiler R package, in which gene length bias was corrected. GO terms with corrected P-value less than 0.05 were considered significantly enriched by differential expressed genes. We used cluster Profiler R package to test the statistical enrichment of differential expression genes in KEGG pathways. A local version of the GSEA analysis tool (http://www.broadinstitute.org/gsea/index.jsp) was used to analyze the GO, KEGG, and Reactome of the species.

**Statistical analyses**

Data are reported as mean±SD. Statistical difference was assessed by unpaired two-tailed Student *t* test. Statistical analyses were conducted using GraphPad Prism software 8.0.1. For all statistical tests, *P* value of <0.05 was considered statistically significance
