# Supplementary material for: Latexin deficiency attenuates adipocyte differentiation and protects mice against obesity and metabolic disorders induced by high-fat diet
Source: Cell Death Dis. 2022 Feb 24;13(2):175. doi: 10.1038/s41419-022-04636-9 (PMC8873487; doi:10.1038/s41419-022-04636-9)
Supplement: Supplementary file 3 — Supplementary Figure and Figure Legends [file 41419_2022_4636_MOESM3_ESM.docx]

**Supplementary Figure and Figure legends**


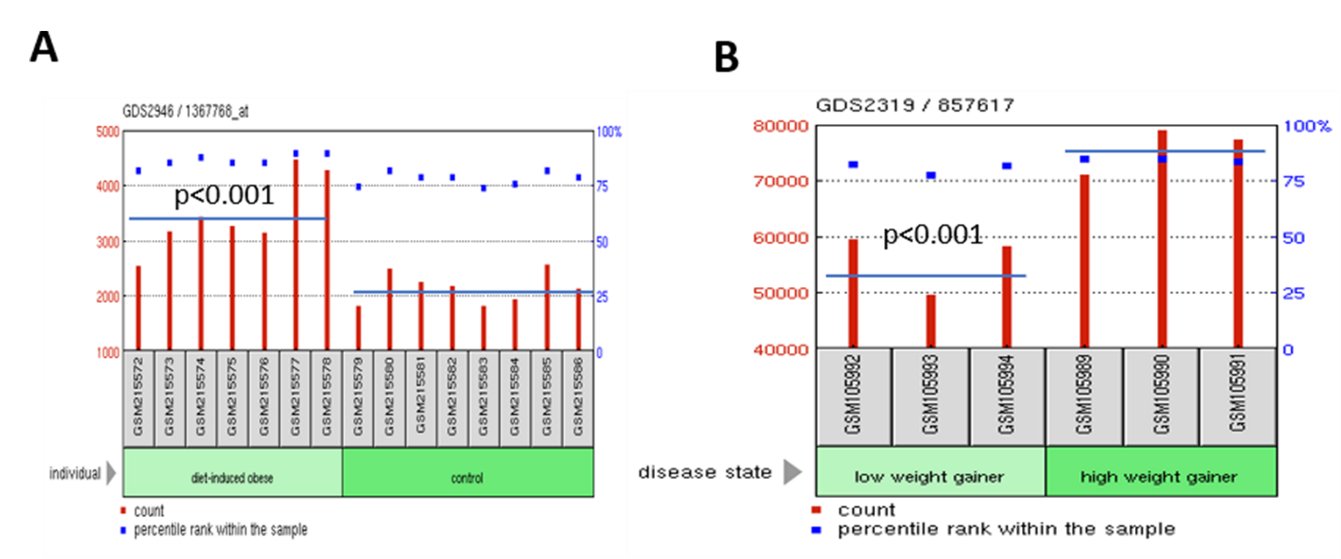


**Fig S1.** Analysis of expression of *LXN* in obese rat (A) and mice (B) tissues based on GEO profile database.


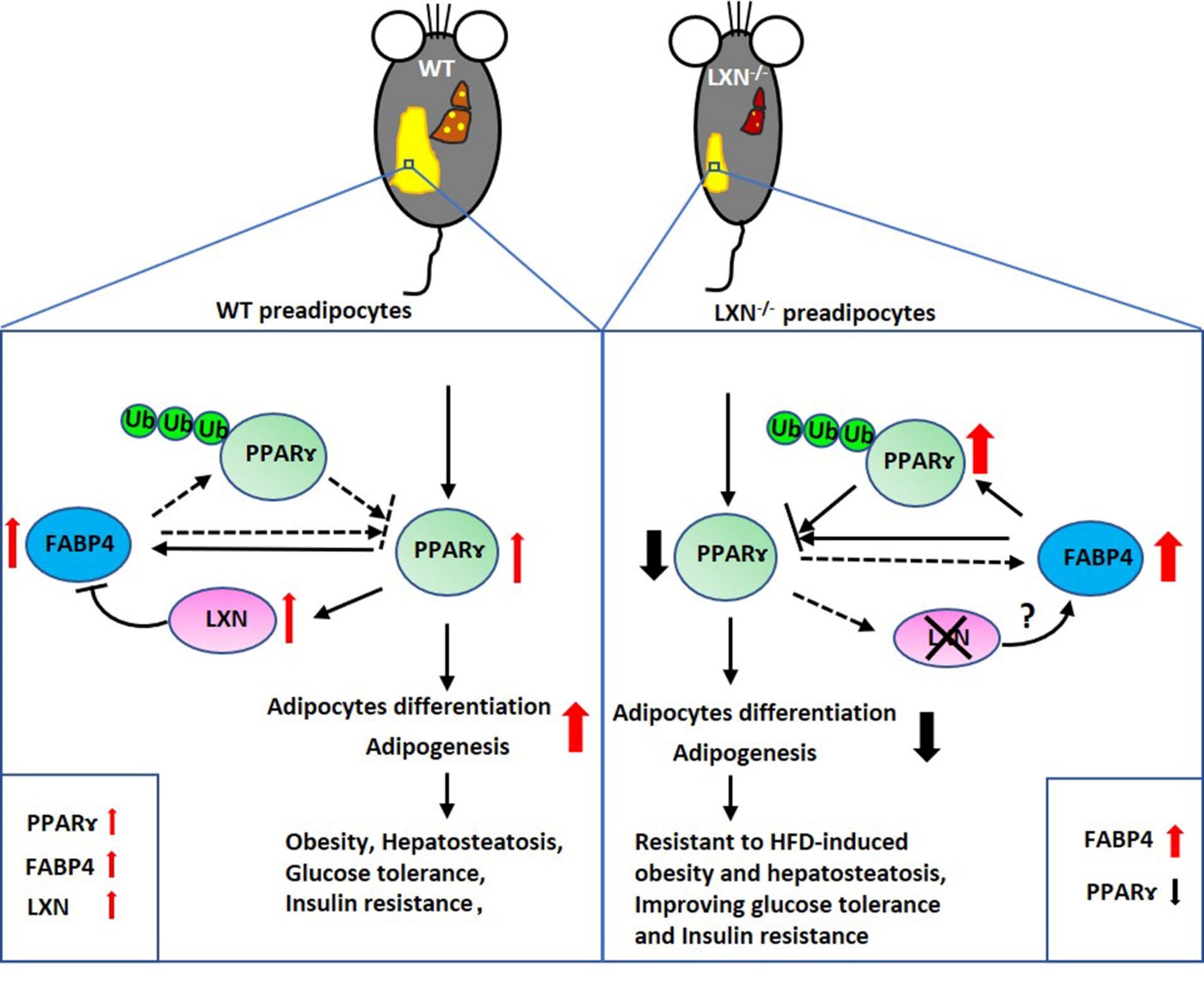


**Fig S2** Schematic diagram to demonstrate the mechanism by which *LXN* loss prevents preadipocytes differentiation and adipogenesis. The protein level of PPARɤ is precisely regulated by LXN and FABP4 during adipocyte differentiation. WT preadipocytes cultured in differentiation medium increased the protein level of PPARɤ, which induced the expression of FABP4 and LXN. Conversely, excessive FABP4 negatively regulates PPARɤ protein level by accelerating its ubiquitination (left, dotted arrow); while LXN positively regulates PPARɤ protein level by inhibiting FABP4 overexpression. In LXN^-/-^ preadipocytes, loss of *LXN* upregulates FABP4 expression via a PPARɤ-independent pathway, resulting in increased PPARɤ ubiquitination (right, solid arrow) and decreased adipocyte differentiation.
