# Supplementary material for: Latexin deficiency attenuates adipocyte differentiation and protects mice against obesity and metabolic disorders induced by high-fat diet
Source: Cell Death Dis. 2022 Feb 24;13(2):175. doi: 10.1038/s41419-022-04636-9 (PMC8873487; doi:10.1038/s41419-022-04636-9)
Supplement: Supplementary file 4 — Supplementary Tables [file 41419_2022_4636_MOESM4_ESM.docx]

**Table S1.** Prediction of transcription factor binding sites in LXN promoter region.

| **Matrix ID** | **Name** | **Score** | **Relative score** | **Sequence ID** | **Start** | **End** | **Strand** | **Predict sequence** |
| --- | --- | --- | --- | --- | --- | --- | --- | --- |
| MA0066.1 | PPARG | 13.934 | 0.813 | LXN promoter | -1634 | -1615 | + | GTAGGTAACCAAGACCCAAG |
| MA0065.2 | Pparg::Rxra | 10.023 | 0.834 | LXN promoter | -2328 | -2314 | + | GAGGGAGAGAGGCGA |
| MA0065.2 | Pparg::Rxra | 9.776 | 0.829 | LXN promoter | -2096 | -2082 | + | GAAGGGCAAAGACTA |
| MA0065.2 | Pparg::Rxra | 9.530 | 0.826 | LXN promoter | -833 | -818 | + | CTGAGGCAGAGGACT |
| MA0065.2 | Pparg::Rxra | 8.338 | 0.806 | LXN promoter | -522 | -508 | + | CAAAGGGAAGGGTAA |

**Table S2** The sequences of nucleotide for qPCR, siRNA

| **For qPCR** | |
| --- | --- |
| **Gene** | **Sequence (5’-3’)** |
| LXN | Forward, 5’-GCGGTTATGTAATGTGGCAG-3’; Reverse, 5’-AATGTCGTGGAGTAGAATGGTG-3’ |
| IL-1β | Forward,5’-TGCCACCTTTTGACAGTGATG-3’; Reverse,5’-TGATGTGCTGCTGCGAGATT-3’ |
| GAPDH | Forward,5’-AGGTCGGTGTGAACGGATTTG-3’; Reverse,5’-TGTAGACCATGTAGTTGAGGTCA-3’ |
| iNOS | Forward, 5’-GTTCTCAGCCCAACAATACAAGA-3’; Reverse, 5’-GTGGACGGGTCGATGTCAC-3’ |
| TNF-α | Forward, 5’-ACCCTCACACTCAGATCATC-3’, Reverse, 5’-GAGTAGACAAGGTACAACCC-3’ |
| FASN | Forward, 5’-TCCAAGACTGACTCGGCTACTGAC-3’, Reverse, 5’-GACGCCAGGTTCGGAATGCTATC-3’ |
| SCD1 | Forward, 5’-TTCTTGCGATACACTCTGGTGC-3’, Reverse, 5’-CGGGATTGAATGTTCTTGTCGT-3’ |
| SCD2 | Forward, 5’-GATCTCTGGCGCTTACTCAGC-3’, Reverse, 5’-CTCCCCAGTGGTGAGAACTC-3’ |
| SCD3 | Forward, 5’-GTTGCCACTTTACTGAGATACGC-3’, Reverse, 5’-GAAGCCCTCGCCCATACTT-3’ |
| PPARɤ | Forward, 5’-GGAAGACCACTCGCATTCCTT-3’, Reverse, 5’-GTAATCAGCAACCATTGGGTCA-3’ |
| FABP4 | Forward, 5’-AAGGTGAAGAGCTACATAACCCT-3’, Reverse, 5’-TCACGCCTTTCATAACACATTCC-3’ |
| CEBPα | Forward, 5’-CTCGCCATGCCGGGAGAACTCTA-3’, Reverse, 5’-GAGGTGACTGCTCATCGGGGGC-3’ |
| **For plasmid construction** | |
| LXN | Forward,5’-CACGAATTCAATGGAAATCCCGCCGA-3’; Reverse,5’- TCTAGAATCCTTCCAGTTGTACTTCC-3’ |
| **siRNA** | |
| LXN | 1#:5’-GAAACGAUGACUUCAUUGAdTdT-3’, 2#: 5’-UCAAUGAAGUCAUCGUUUCdTdT-3’ |

**Table S3 The antibodies for Western blot**

| **Name** | **Supplier** | **Catalog** | **WB** | **IF** | **IHC** | **IP** |
| --- | --- | --- | --- | --- | --- | --- |
| **LXN** | **Sino Biological** | **10211-R101** | **1:1000** |  | **1:100** |  |
| **GAPDH** | **ZSGB-BIO** | **TA-08** | **1:1000** |  |  |  |
| **β-actin** | **ZSGB-BIO** | **TA-09** | **1:1000** |  |  |  |
| **IgG** | **Beyotime** | **A7028** |  |  |  | **2 µg/mg of lysate** |
| **Flag** | **Sino Biological** | **101274-MM05** | **1:1000** |  |  |  |
| **F4/80 Antibody, eFluor 570** | **Thermo Scientific** | **41-4801-82** |  | **5 µg/mL** |  |  |
| **p-mTOR(Ser2481)** | **Abcam** | **Ab137133** | **1:1000** |  |  |  |
| **mTOR** | **Proteintech** | **66888-1-1g** | **1:1000** |  |  |  |
| **PPARɤ** | **Abcam** | **Ab41928** | **1:1000** |  |  | **2 µg/mg of lysate** |
| **FABP4** | **Proteintech** | **17509-1-AP** | **1:1000** |  |  |  |
| **p-AKT (Ser473)** | **Proteintech** | **66444-1-1g** | **1:1000** |  |  |  |
| **AKT** | **Proteintech** | **10176-2-AP** | **1:1000** |  |  |  |
| **p-IRS (Ser307)** | **Abcam** | **Ab5599** | **1 µg/mL** |  |  |  |
| **IRS1** | **Abcam** | **Ab245314** | **1:2000** |  |  |  |
| **Ub** | **Abcam** | **Ab33893** | **1:1000** |  |  |  |
| **DyLight680 conjugated** **Anti-rabbit IgG (H+ L)** | **Thermo Scientific** | **35568** | **1:10000** |  |  |  |
| **DyLight 800 conjugated** **Anti-mouse IgG (H+L)** | **Thermo Scientific** | **SA5-35521** | **1:10000** |  |  |  |
