# Supplementary material for: Latexin deficiency attenuates adipocyte differentiation and protects mice against obesity and metabolic disorders induced by high-fat diet
Source: Cell Death Dis. 2022 Feb 24;13(2):175. doi: 10.1038/s41419-022-04636-9 (PMC8873487; doi:10.1038/s41419-022-04636-9)

Uncropped scans of membranes and gels used in the main figures.


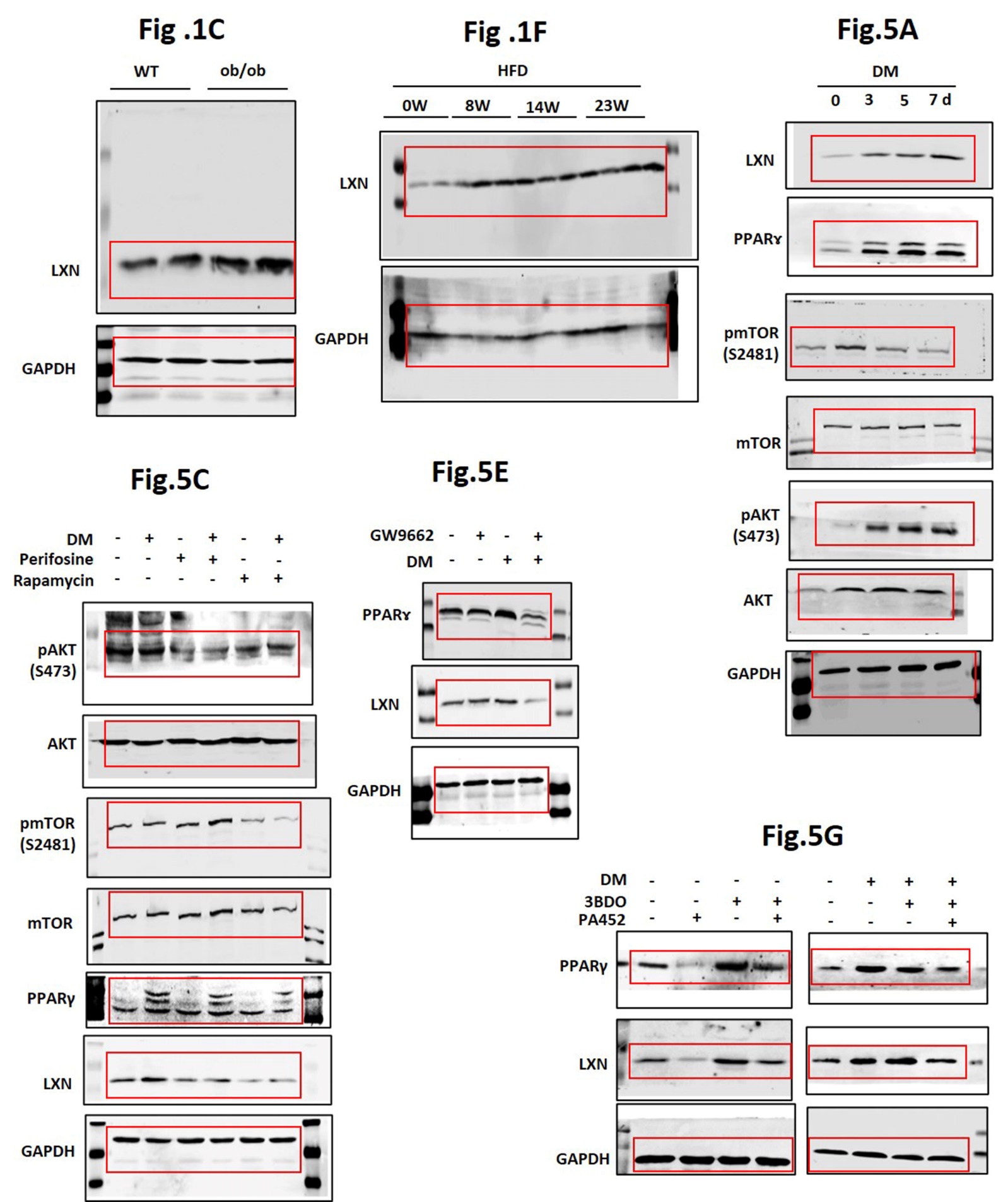


Uncropped scans of membranes and gels used in the main figures- Continued


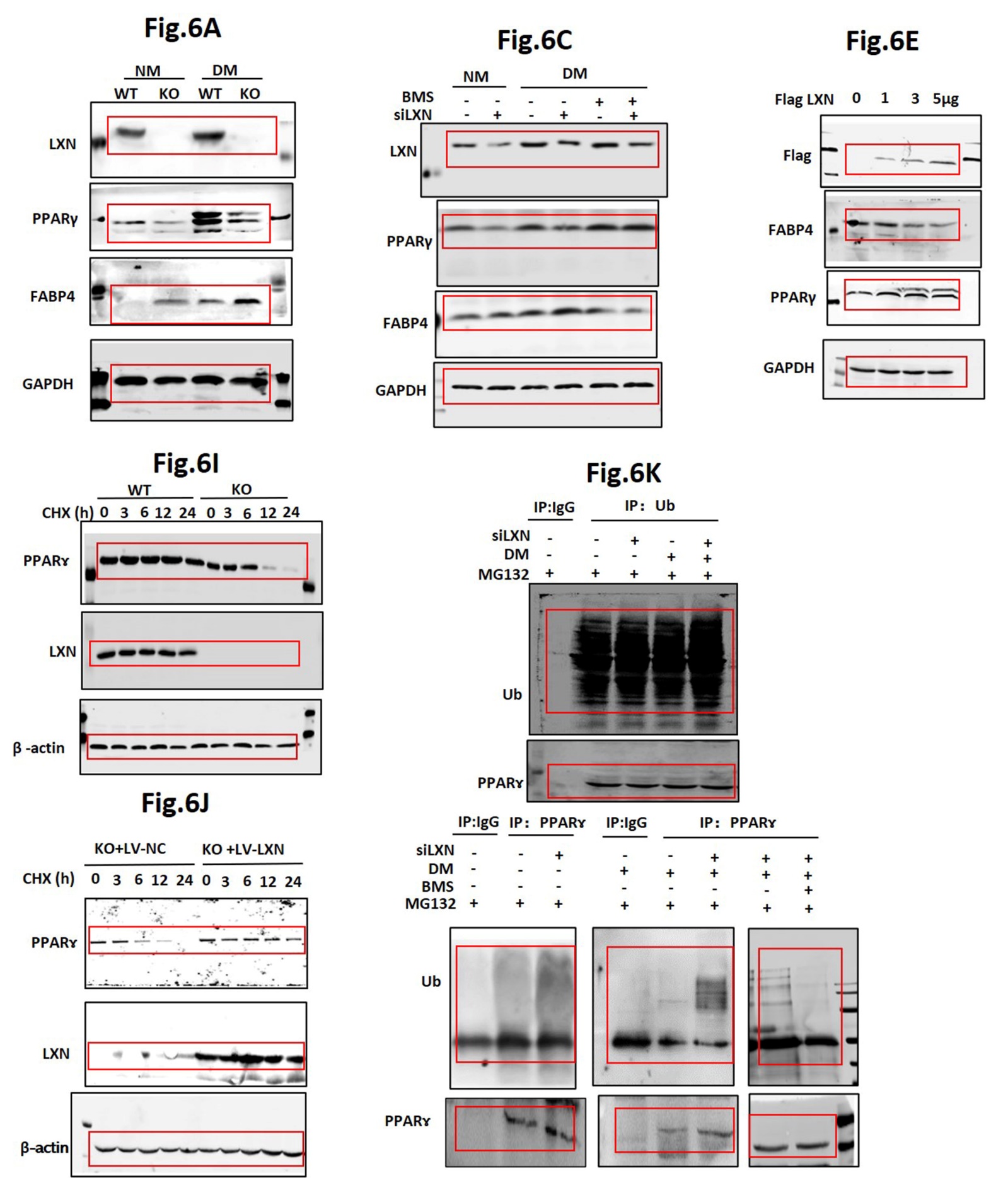


Uncropped scans of membranes and gels used in the main figures- Continued


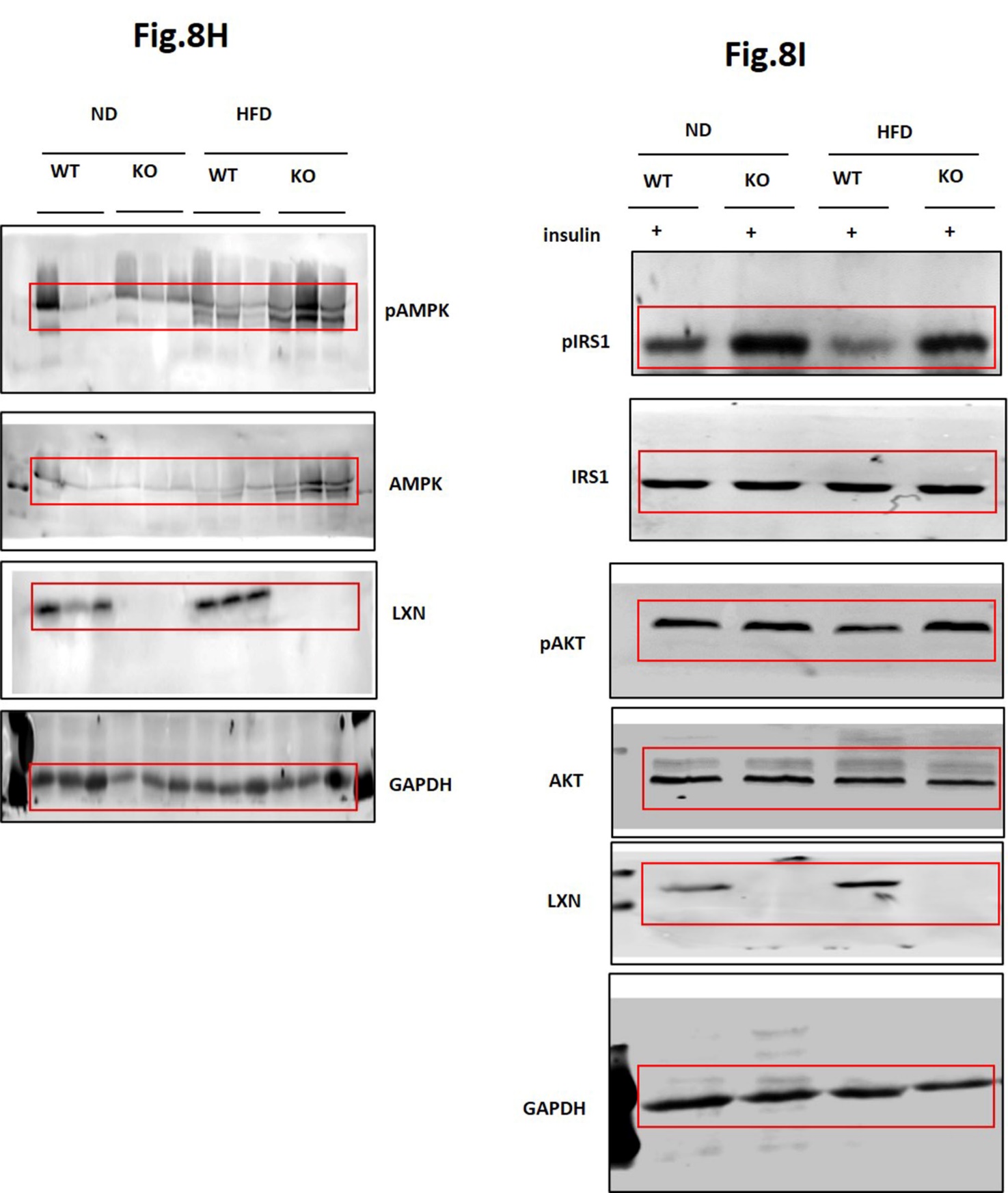

Supplement: Supplementary file 5 — Uncropped western blot [file 41419_2022_4636_MOESM5_ESM.docx]
